# Loss of miR-144 signaling interrupts extracellular matrix remodeling after myocardial infarction leading to worsened cardiac function

**DOI:** 10.1038/s41598-018-35314-6

**Published:** 2018-11-15

**Authors:** Quan He, Fangfei Wang, Takashi Honda, Jeanne James, Jing Li, Andrew Redington

**Affiliations:** 1 The Heart Institute, Cincinnati Children’s Hospital Medical Center, Cincinnati, Ohio, USA; 2 Division of Cardiology, Labatt Family Heart Center, Hospital for Sick Children, Toronto, Ontario, Canada

## Abstract

We have previously shown that MicroRNA (miR) -144 is a key modulator of the acute cardioprotection associated with remote ischemic preconditioning and post myocardial infarction (MI) remodeling. In this study we examine the biology of the remodeling response after permanent ligation of the left anterior descending coronary artery in male miR-144 KO mice, and wild-type littermates (WT). Collagen content and cross linking were determined by hydroxyproline and pyridinoline assays, MI size and scar thickness were measured post PicoSirius Red staining, and cardiac function was evaluated by echocardiography. miR-144 KO mice developed normally with normal cardiac function, however after MI, infarction size was greater and scar thickness was reduced in miR-144 KO mice compared with WT littermates. miR-144 KO mice had a lower incidence of acute cardiac rupture compared with WT littermates early after MI but there was impaired late remodeling, reflected by increased total cardiac collagen content and collagen cross-linkage associated with changes in Zeb1/LOX1 axis, and decreased left ventricular ejection fraction. We conclude that miR-144 is involved in extracellular matrix remodeling post MI and its loss leads to increased myocardial fibrosis and impaired functional recovery.

## Introduction

Cardiac extracellular matrix (ECM) is a three dimensional dynamic structure composed of a basement membrane, interstitial matrix and extracellular fluid. The extracellular fluid distributes its contents throughout the ECM so that soluble proteins, hormones, nutrients, ions, and signaling molecules can be delivered to their targets. The structure of ECM is composed of proteoglycans, glycoproteins, cell-matrix interaction proteins, and collagens, which are the major structural components of the ECM. Blood vessels and capillaries, cardiac myocytes, fibroblasts, and other cardiac resident cells are all organized within ECM, but fibroblasts, macrophages, lymphocytes, and mast cells are migratory within the ECM, responding to local stress and injury. Indeed, in the event of myocardial infarction (MI), local cardiomyocyte cell death leads to diffuse inflammatory cell invasion and activation of fibroblasts leading to fibroblast proliferation and transactivation to myofibroblasts^1^. Myofibroblasts are characterized by their expression of α-smooth muscle actin and enhanced collagen synthesis and secretion, and are primarily responsible for collagen-rich replacement scar formation that occurs after infarction.

Collagen biosynthesis and maturation is a complex process composed of several steps including translation and post translational modification (hydroxylation and glycosylation) of three procollagen chains folding into a triple helix, multiple triple helix self-assembly, and inter triple helix cross-linking catalyzed by lysyle oxidases (LOX)^2^. LOX catalyze the –NH2 of telopeptidyl-lysine and hydroxylysine to –COH, the –COH group then reacts with –NH2 from adjacent telopeptidyl-lysine or telopeptidyl-hydroxylysine to form double-covalent cross linking, which subsequently undergo triple-covalent cross linkage by pyridinolines, via non-enzymatic reactions^3^. The primary sequence of collagens contains special Glycine-X-Y repeats, where X and Y can be any amino acids but more often are proline and hydroxyproline. Hydroxyproline, a product of post-translational modification, is almost exclusively present in collagen and accounts for 13.5% of collagen amino acids^4^. Pyridinoline and hydroxyproline contents are key targets for collagen cross linking and concentration. As the major structural protein in cardiac ECM, collagens have significant impact on cardiac remodeling post MI, not only in respect of their concentration but also their cross linking^5^. It is well known that enhanced collagen deposition is associated with myocardial fibrosis^6^, and enhanced collagen cross linking results in stronger post-infarct scar and reduced left ventricular myocardial distensibility, in turn leading to cardiac dysfunction^7^. Clinical data also show that increased collagen cross-linking is associated with elevated filling pressure^8^ and increased likelihood of heart failure hospitalization^9^.

MicroRNAs (miRNA) are a large class of short (about 22nt) endogenous noncoding RNAs. They regulate target protein levels post-translationally via binding to their partially complementary sequences (binding sites) in the untranslated region leading to mRNA degradation and/or stop translation. Dysregulation of miRNAs has been shown to be involved in many human diseases including (but not limited to) cardiovascular disease^10^, neurodegenerative diseases^11^, and cancers^12^. We have previously shown that miR-144 is a key modulator of the acute cardioprotection associated with remote ischemic preconditioning. Furthermore, we have recently shown that miR-144 loss-of-function increases, and miR-144 supplementation reduces, post myocardial infarction remodeling^13,14^. However, the underlying biology of these responses remains incompletely understood, particularly in terms of the mechanisms driving the relationship between loss of function and adverse remodeling. The current studies were therefore performed in miR-144/451 knockout (subsequently referred to as miR-144 KO) mice, using a permanent ligation model of myocardial infarction, to obviate any confounding effects on reperfusion injury. We first characterized global functional responses in the miR-144 KO strain and then examined the acute and chronic structural and functional responses to myocardial infarction.

## Results

### Characterization of miR-144 KO mice

**Figure 1:**
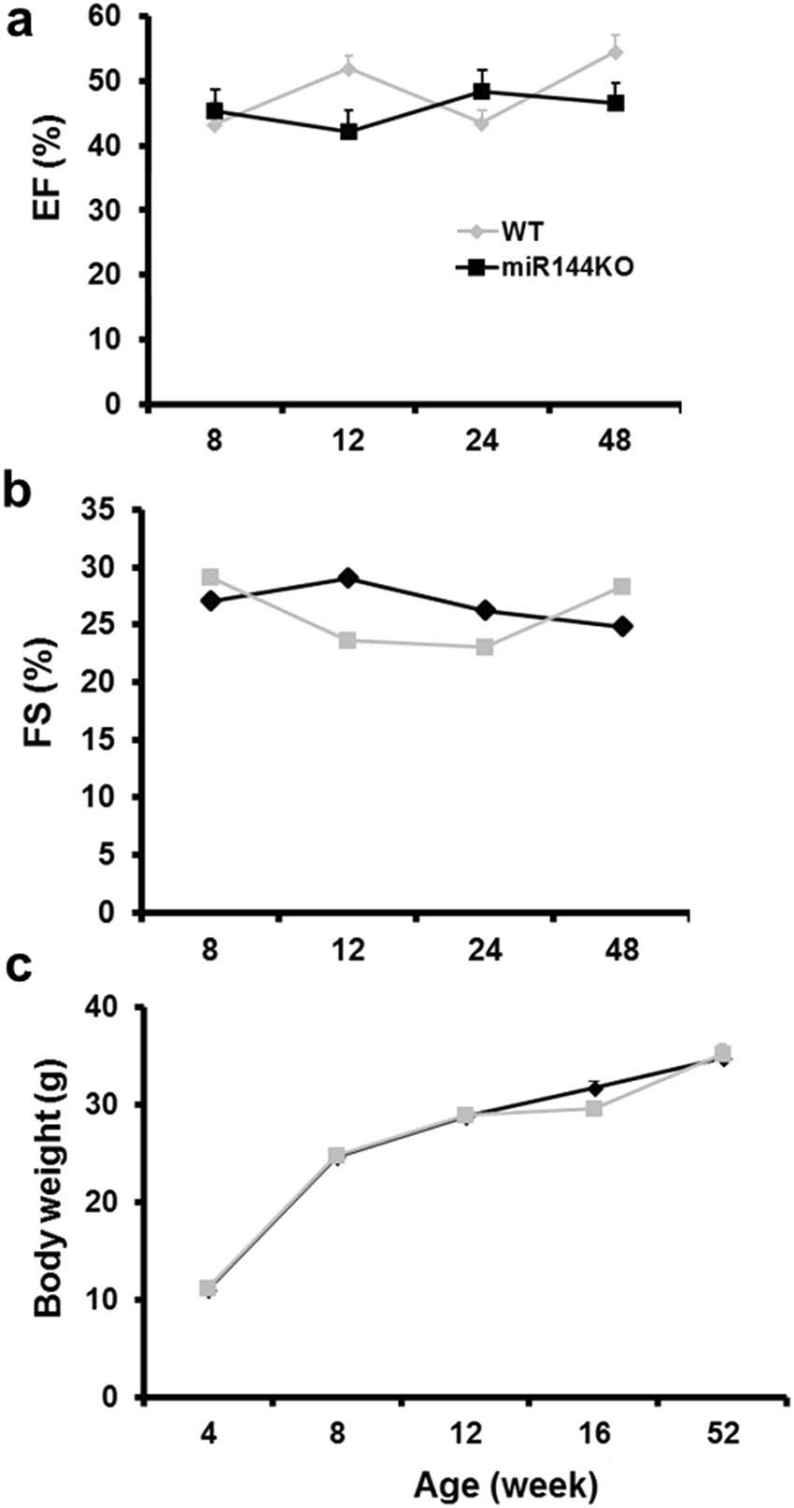
miR-144 KO mice develop normally. Cardiac function was represented by ejection fraction (**a**) and fraction shortening (**b**). N = 7 for both WT and miR-144. (**c**) Growth curve. For the WT group N = 17, 10, 12, 11, and 5 for age 4, 8, 12, 16, and 52 weeks accordingly, while N = 11, 10, 10, 12, and 14 for the KO group in the corresponding age time points.

Consistent with previous reports^15^, miR-144 KO mice appeared to develop normally. As shown in Fig. 1a,b, cardiac function represented by ejection fraction (EF) and fractional shortening (FS) is not different between miR-144 KO and wild type littermates up to 48 weeks old. miR-144 KO’s also had normal somatic growth (Fig. 1c).

### miR-144 KO interferes with scar formation post MI

**Figure 2:**
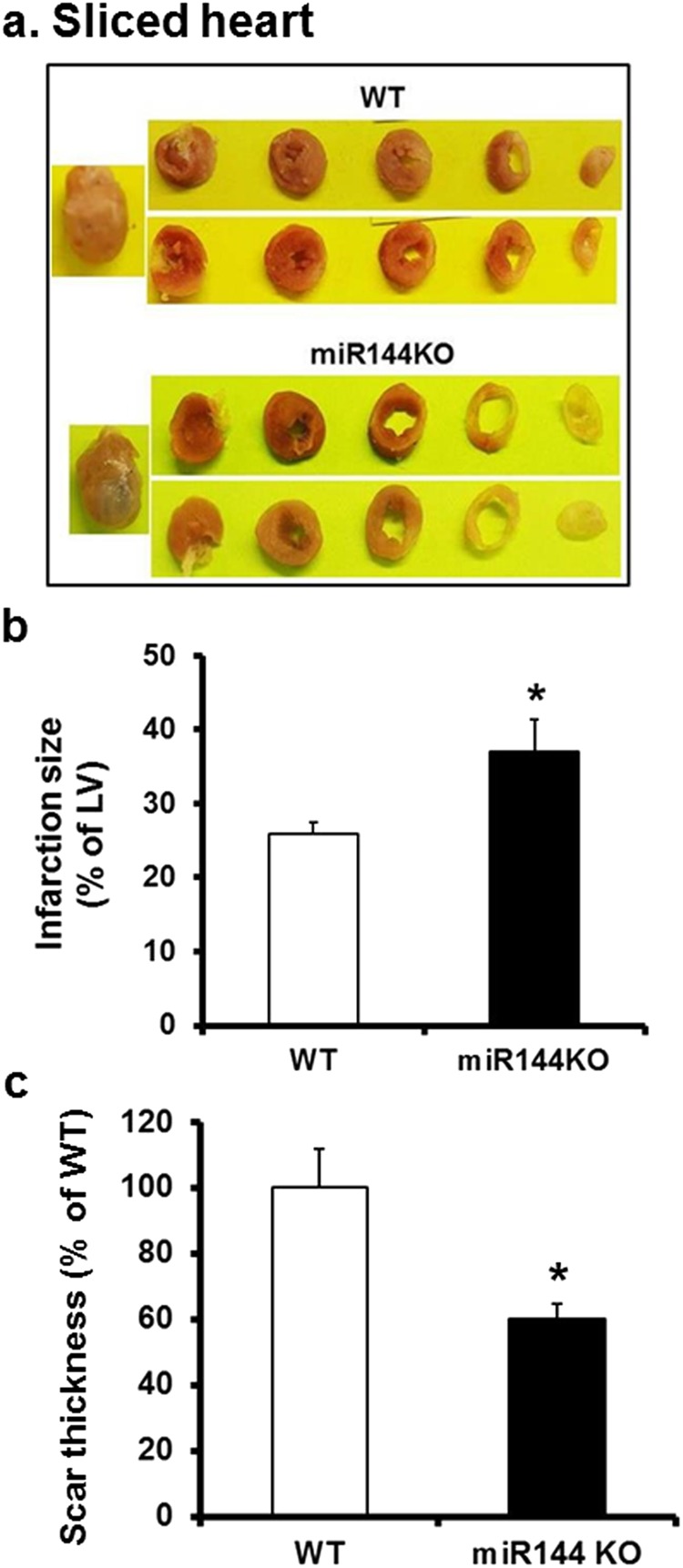
Coronary artery ligation induces larger but thinner scar in miR-144 KO mice. (**a**) Sliced heart. Both Infarction size (**b**) and scar thickness (**c**) were determined from images of the PicroSirius stained slides as detailed in the Methods. *p < 0.05 compared with WT, N = 7 for WT and 6 for miR-144 KO.

As ECM remodeling greatly affects scar formation after myocardial infarction, we next evaluated the scar formation in miR-144 KO mice subjected to left anterior descending coronary artery occlusion. As shown in Fig. 2a, miR-144 KO was associated with larger MI size and thinner scar thickness as compared with WT littermates 4 weeks post MI. Overall, the MI size of miR-144 KO was increased by approximately 30%, and scar thickness decreased 40% compared with WT littermates (Fig. 2b,c).

### miR-144 KO increases collagen concentration and cross-linking

**Figure 3:**
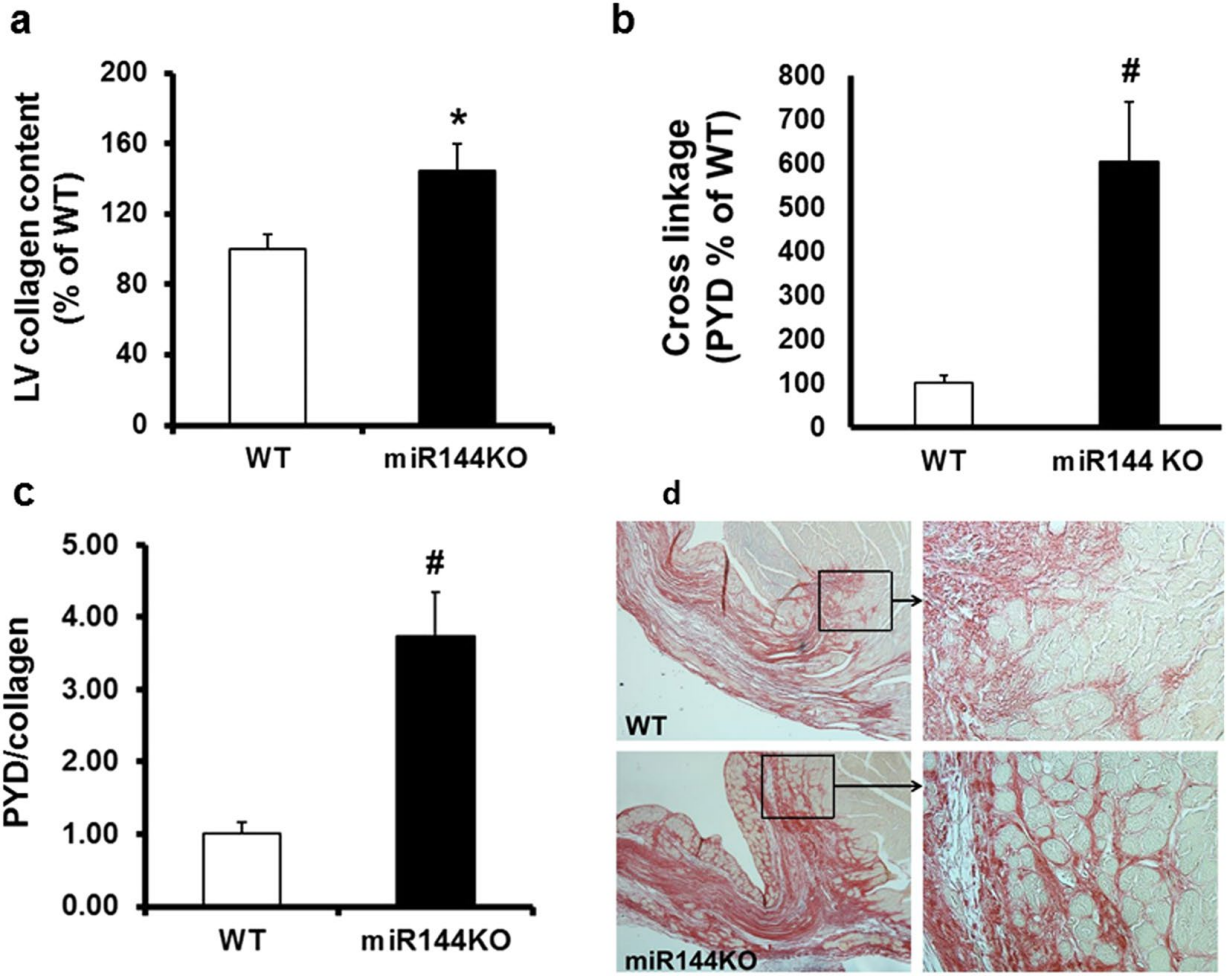
miR-144 KO modifies ECM remodeling post MI. (**a**) Left ventricular collagen contents. *P < 0.05 compared with WT, N = 6 for WT and 7 for miR-144 KO. (**b**). Collagen cross linkage. ^#^p < 0.01 compared with WT, N = 6 for WT and 7 for miR-144 KO. (**c**) Collagen cross linkage corrected by collagen content. ^#^p < 0.01 compared with WT, N = 6 for WT and 7 for miR-144 KO. (**d**) Collagen networks. Pictures are representive of images from 7 mice in the WT group and 6 mice in the miR-144 KO group.

We next examined if the adverse scar formation influences left ventricular ECM remodeling. Not only was left ventricle collagen content increased by approximately 45% in miR-144 KO, compared with WT littermates (as measured by hydroxyproline assay - Fig. 3a), but collagen cross linkage was greatly increased, by approximately 6-fold (Fig. 3b). The increased collagen cross-linking cannot be explained by the increased collagen content as the cross linkage was still increased almost 4-fold compared with WT littermates, after correcting for the collagen content increase (Fig. 3c). Consistent with the increased collagen cross-linking, miR-144 KO hearts also displayed better developed collagen networks, especially in the border zone (Fig. 3d) compared with WT littermates.

### LOX1 was increased in miR-144 KO mice

**Figure 4:**
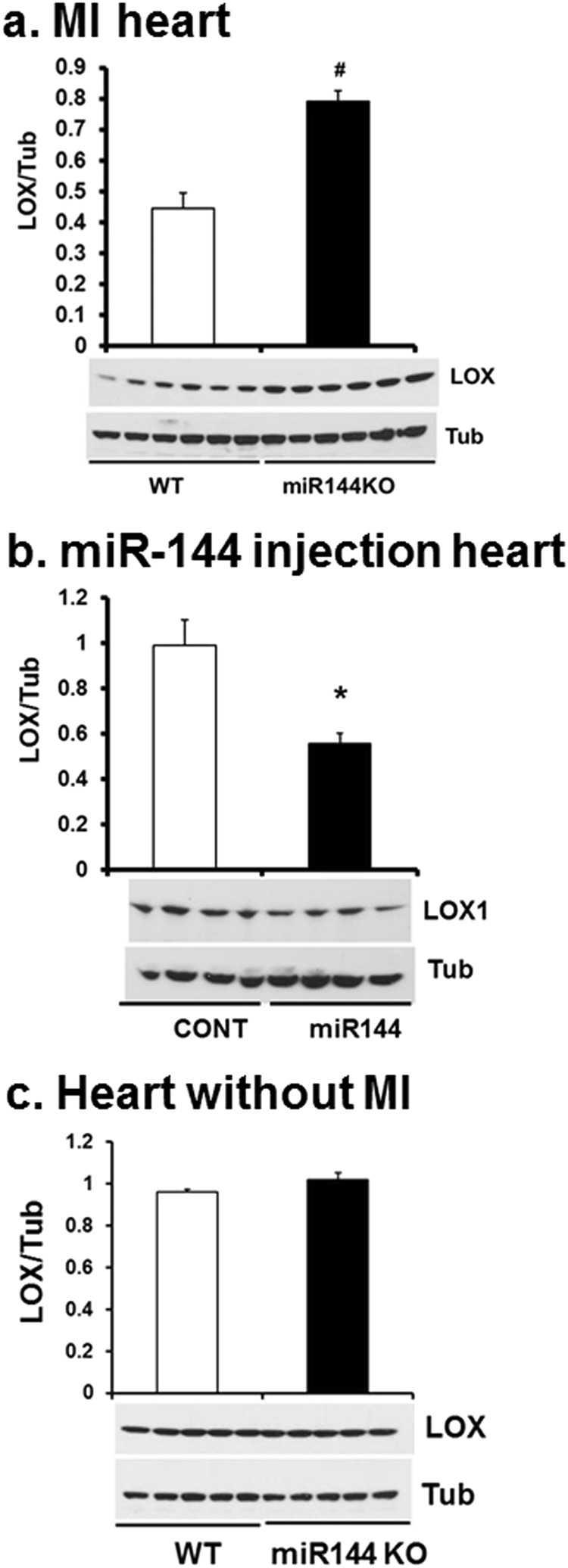
miR-144 knocks down LOX1 protein levels. LOX1 protein levels were determined by Western blot from the hearts of mice which underwent MI 7 days previously (**a**). Cardiac LOX1 levels were also determined from mice injected with miR-144 (**b**) and miR-144 KO mice without MI (**c**). *p < 0.05 and ^#^p < 0.01 compared with WT or CONT which is PBS injection. N = 6 for WT and miR-144 KO in panel a; N = 4 for both miR-144 and PBS injection in panel b; and N = 5 for both WT and miR-144 KO in panel c.

LOX proteins catalyze the formation of collagen cross linkage^2^. We therefore tested if LOX proteins were induced differentially in miR-144 KO hearts post MI. We found that LOX1 was upregulated in miR-144 KO hearts (Fig. 4a) and tail vein injection of miR-144 decreased cardiac LOX1 protein (Fig. 4b). Cardiac LOX1 protein levels were similar between miR-144 KO and WT littermates without MI (Fig. 4c) implying that miR-144 may be regulating infarct responses rather than basal activity of LOX. We failed to find miR-144 targets in LOX transcripts suggesting that miR-144 regulates LOX1 indirectly. Full blots are provided for all data in the supplementary data section.

### The Zeb1/LOX axis is a target of miR-144

**Figure 5:**
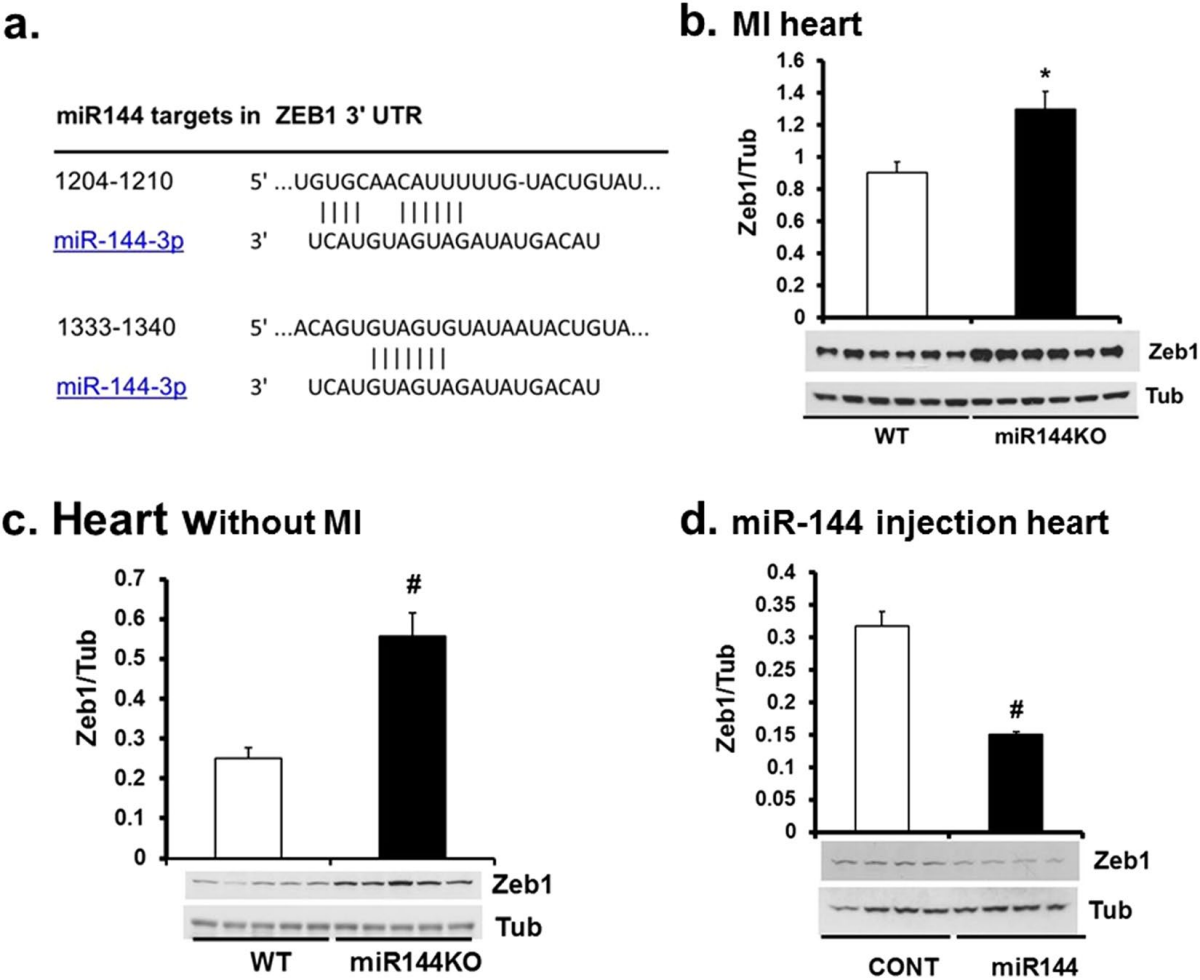
miR-144 targets Zeb1 in the heart. (**a**) miR-144 targets in Zeb1 mRNA untranslated region. Cardiac Zeb1 Protein levels were determined by Western blot from WT mice after myocardial infarction (**b**), miR-144 KO mice without MI (**c**), and miR-144 injection (**d**). *p < 0.05 and ^#^p < 0.01 compared with WT or CONT which is PBS injection. N = 6 for WT and miR-144 KO in panel b; N = 5 for both miR-144 KO and WT in panel c; and N = 4 for both miR-144 injection and CONT in panel d.

**Figure 6:**
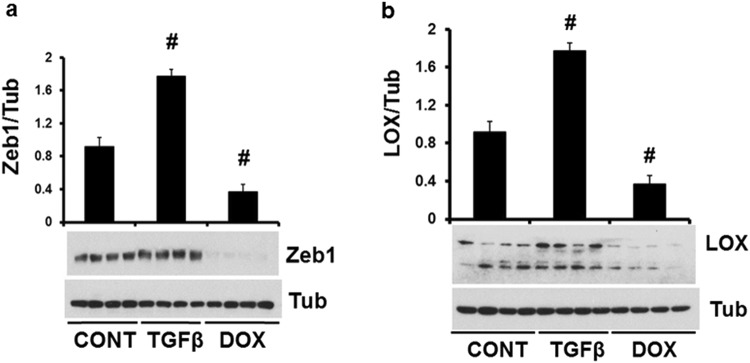
LOX1 is positively correlated with Zeb1 in cultured neonatal cardiac fibroblasts. Both Zeb1 (**a**) and LOX1 (**b**) were induced by TGFβ but suppressed by DOXO (doxorubicin). ^#^p < 0.01 compared with CONT, N = 4 for both panel a and b.

Zeb1 regulates LOX in cancer cells^16^. We found 2 highly conserved miR-144 targets in Zeb1 transcripts (Fig. 5a) but we failed to find any miR-451 targets. Zeb1 was significantly induced in miR-144 KO heart post MI compared with WT littermates (Fig. 5b). Further experiments confirmed that basal Zeb1 was also increased in miR-144 KO hearts but was decreased in miR-144 injected mouse hearts (Fig. 5c,d) suggesting that Zeb1 is a potential target of miR-144. To confirm the existence of Zeb1/LOX axis function in cardiac cells, we studied cultured neonatal cardiac fibroblasts. As shown in Fig. 6a, Zeb1 was induced by TGF1β but was repressed by doxorubicin (DOXO). The responses of LOX1 to these treatments followed the same pattern as Zeb1 (Fig. 6b). Zeb1 and LOX1 expression was positively correlated in cultured neonatal cardiac fibroblasts. This is consistent with previous reports that TGF1β induces but DOXO reduces expression of Zeb1^17,18^. We conclude that the Zeb1/LOX axis is a biologically significant target of miR-144 in the heart.

### Diffusely enhanced collagen cross linking is characteristic of post-MI remodeling in miR144 KO myocardium

**Figure 7:**
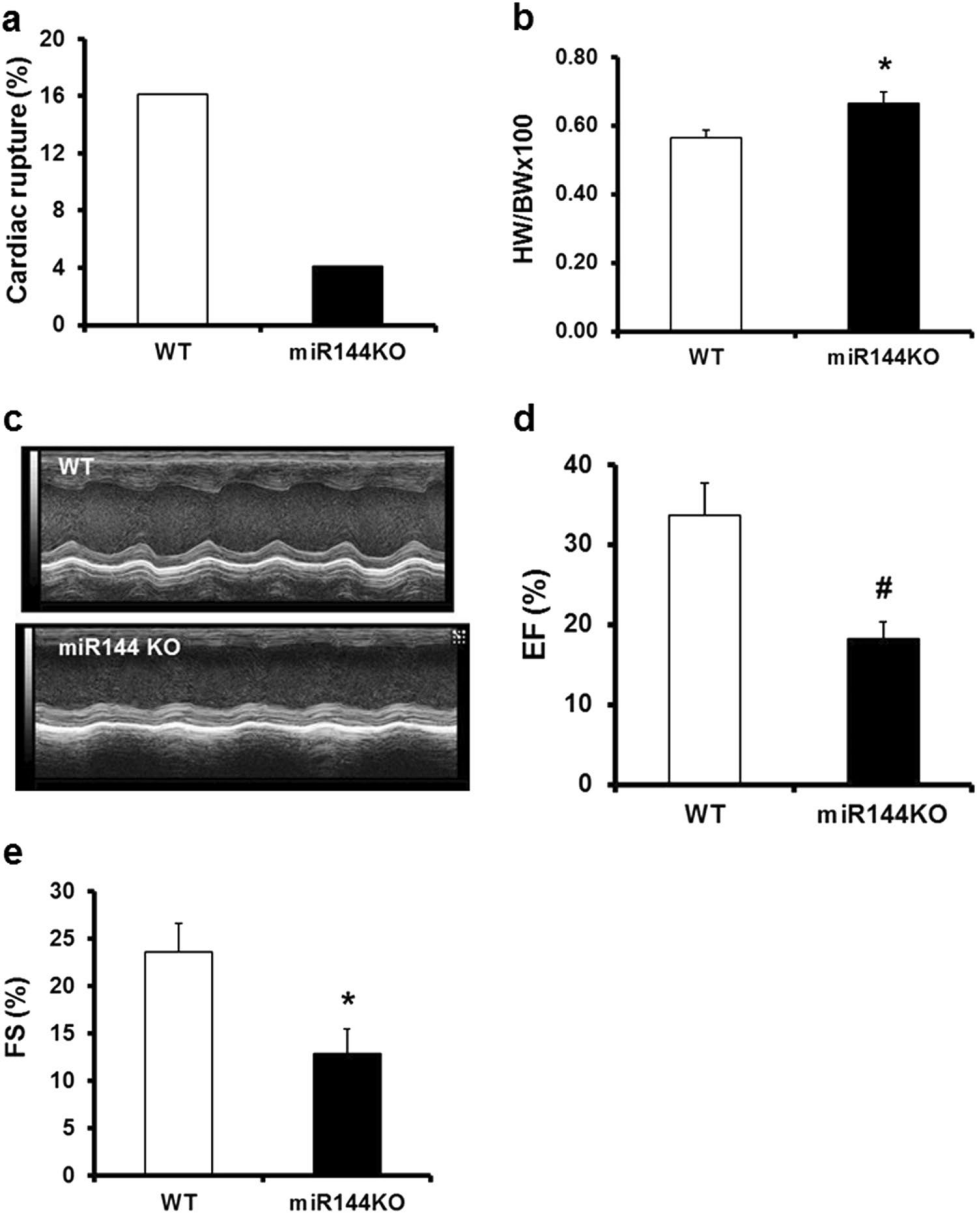
miR-144 KO interferes with the healing process post MI leading to worsened cardiac function. (**a**) Acute cardiac rupture post MI. N = 31 for WT and 49 for miR-144 KO. (**b**) Heart and body weight ratio. *p < 0.05 compared with WT. N = 13 for both WT and miR-144 KO. (**c**) Represent images of echocardiography. (**d**) Ejection fraction. ^#^p < 0.01 compared with WT. N = 13 for WT and miR-144 KO. (**e**) Fraction shortening. *p < 0.05 compared with WT. N = 13 for WT and miR-144.

The enhanced propensity to collagen cross-linking may have far reaching impact on the heart after myocardial infarction. Interestingly, despite thinner scar thickness, the early cardiac rupture rate was much lower in miR-144 KO mice compared with their WT littermates (WT 16% vs 4% miR-144 KO, Fig. 7a). However, the enhanced collagen cross-linking also adversely affected the chronic adaptation process post MI. The heart weight - body weight ratio was increased in miR-144 KO mice compared with WT littermates (Fig. 7b), and miR-144 KO hearts displayed decreased contractility (Fig. 7c). Global cardiac function was also significantly decreased as indicated by the decline of EF and FS in miR-144 KO mice compared with WT littermates (Fig. 7d,e). The characteristics of chronic remodeling in miR-144 KO hearts appears different from WT littermates as MI size was strongly correlated with EF (R^2^ = 0.789) in WT littermates but not in miR-144 KO mice (R^2^ = 0.001).

## Discussion

Building on our prior studies showing that miR-144 plays a potential role in acute cardioprotection^13^, and improving post MI remodeling^14^ after ischemia-reperfusion injury, this study was designed to examine the mechanisms involved in adverse remodeling associated with loss of miR-144 function. At basal conditions, loss of miR-144 does not affect the animals overtly, somatic growth was normal, and myocardial performance was unaffected during an almost 1 year period of monitoring. However, loss of miR144 signaling after MI is associated with significant differences in myocardial responses to injury. These differences are manifest locally as abnormalities of post MI scar formation and maturation, and more diffusely as abnormalities of myocardial collagen deposition and associated adverse cardiac remodeling.

The process of post-MI healing starts almost immediately after the onset of the MI itself^19^. MI induced cardiac myocyte death via apoptosis and/or necrosis activates a variable degree of inflammatory cell and fibroblast activation. This process has been reviewed extensively^19,20^, but suffice it to say that while this is a tightly regulated cascade, relatively small perturbations that modify the degree of inflammatory response can have major effects on the subsequent structural and functional myocardial responses (remodeling), even when infarct size itself is relatively constant. Indeed, experimental modification of the inflammatory response after MI can significantly improve adverse post-MI remodeling^14,21,22^. There is a fine balance between the appropriate local inflammatory and fibrotic responses that lead to beneficial infarct healing (by replacement fibrosis)^23^, and the same processes occurring more remote to the infarcted area, that can be adverse (reactive fibrosis)^24^. Replacement fibrosis is, of course, pivotal for scar formation in the infarcted area, and is responsible for maintaining the structural integrity of the myocardium. Indeed, inadequate scar tensile strength can lead to cardiac rupture in the early post-MI period^25^. Conversely, reactive fibrosis in the peri-infarct area, and more remotely in unaffected myocardium, is maladaptive, leading to adverse remodeling with decreased left ventricular function and ultimately heart failure^26,27^. Our data show that miR-144 and its downstream signaling plays a role in both replacement and reactive fibrosis. Interestingly, although miR-144 KO mice had increased infarct size and thinner scar (Fig. 2), they had increased collagen cross-linking (Fig. 3c) and better collagen networks (Fig. 3d) compared to wild-type mice. These changes were associated with a reduced incidence of cardiac rupture early after MI (Fig. 7a). Furthermore, we identified novel changes in miR-144 targets Zeb1/LOX1 axis which may be related to these differences in collagen cross-linking modification. Zeb1 has been shown to increase collagen I and III gene expression^16^ but, as is usual for micro-RNA’s, miR-144 has many regulatory targets. There is an extensive literature regarding its impact on, for example, IRS1 (insulin receptor substrate 1)^28^, SMAD4^29^, E2F3^30^, c-MET^31^, Epidermal growth factor receptor^32^, and TIGA (The TP53-inducible glycolysis and apoptosis regulator)^33^ in cancer biology; Notch1, PTEN (phosphatase and tensin homolog), and SPRED (sprouty-related suppressor of Ras signaling) in neurocognitive regulationy^34^; and ABCA1 (adenosine triphosphate-binding cassette transporter A1)^35^ and Rac1^15^ in the cardiovascular system. Our previous studies showed that the mTOR-autophagy pathway was involved in the early and late cardioprotection of miR-144 when delivered exogenously^13,14^. Not studied in those prior papers, we are the first to identify the Zeb1/LOX1 pathway as a miR-144 target. We found two miR-144 targets in 3′ untranslated region of Zeb1 via TargetScan (http://www.targetscan.org/vert_71) and demonstrated that Zeb1 was increased in miR-144 KO mouse (Fig. 5c) and substantially decreased after miR-144 administration via tail vein injection (Fig. 5d). These data are compatible with prior findings, in separate studies, that myocardial ischemia reduces miR-144 levels^13^,and increased Zeb1 expression is protective in neurons subjected to ischemic damage^36^. Expression of LOX1 was positively correlated with Zeb1 after MI, although interestingly it was not increased in miR-144 KO mouse hearts suggesting a complementary mechanism for its basal regulation exists, as has been suggested previously^37^. It is also known that TGFβ induces Zeb1 but doxorubicin reduces its expression in tumors^17,18^. We therefore examined the Zeb1/LOX1 pathway in cultured neonatal cardiac fibroblasts, and showed that LOX1 and Zeb1 were similarly up-regulated by TGFβ and down-regulated by doxorubicin (Fig. 6). Thus we have demonstrated a plausible inverse relationship between miR-144 expression and the degree of collagen cross-linking and scar integrity that impacts on the frequency of early post-MI myocardial rupture.

This potential benefit of decreased miR-144 signaling on replacement fibrosis and scar integrity is offset by our findings in terms of reactive fibrosis and later post-MI remodeling, where reduced miR-144 signaling appears to have an adverse effect. There was increased collagen content, and the heart weight/body weight ratio was increased in miR-144 KO mice (Fig. 7b). Furthermore, knockouts had significantly worse cardiac function indicated by decreased EF and FS (Fig. 7d,e). The mechanism for this maladaptive response is unclear.

As we have discussed, our data show a putative role for Zeb1/LOX1 in terms of replacement fibrosis, however the role of Zeb1/LOX1 in the myocardium has otherwise not been studied. Further studies to examine their role in post-MI myocardial fibrosis and remodeling would be worthwhile, as would translational studies to examine how miR-144 expression itself might modify recovery in patients after myocardial infarction. However, it should be noted that our model of permanent ligation may not be entirely relevant to patients that have ischemia-reperfusion injury after thrombolysis or coronary intervention^38^. This may be important as increased collagen cross-linking is associated with increased filling pressure and a higher risk for subsequent heart failure in hypertensive patients^8,9^. Clinical studies may help to understand the potential impact of miR-144 on post-MI responses. Circulating levels of miR-144, interleukins, and stromal cell-derived factor-1 (SDF-1) are potential biomarkers of the responses. The latter is particularly interesting as the cardioprotective SDF-1/cxc chemokine receptor4 (CXCR4) axis has multiple roles in myocardial infarction including recruitment of vascular stem/progenitor cell, angiogenesis and anti-apoptosis^39^. Zeb1 suppresses CXCR4 in human cardiac mesenchymal progenitor cells^17^. Finally, nuclear factor-κB (NFκB) represents a family of inducible transcriptional factors that regulate a large array of genes involved in inflammation, development, and disease progression^40^. Zeb1 increases NFκB expression^41^, as well as interleukin-6 and 8^42^, circulating levels of which are highly correlated with clinical outcomes after myocardial infarction^43^.

In summary, loss of miR-144 worsens post-MI remodeling. We identified a miR-144 target, the Zeb/LOX pathway, which contributes to maladaptive ECM remodeling post MI. Activation of this pathway in miR-144 KO mice leads to increased collagen cross linkage, reducing the incidence of cardiac rupture in the acute phase of MI, but worsening cardiac function during chronic adaptation. We conclude that miR-144 plays a potentially important role in ECM remodeling after myocardial infarction.

## Material and Methods

### Materials

Pierce BCA Protein Assay Kit and ECL Western Blotting Substrate, Restore™ Western Blot Stripping Buffer, PVDF membrane and HRP-conjugated anti-rabbit IgG were ordered from Thermo Fisher Scientific (Rockford, IL). Primary antibodies against Zeb1 and α-tubulin were purchased from Cell Signaling technology (Boston, MA). Primary antibody against LOX1 was obtained from Novus Biologicals (Littleton, CO), the hydroxyproline colorimetric assay kit was from BioVision (Milpitas, CA), and the mouse pyridinoline Elisa kit from MyBioSource (San Diego, CA). Modified miR-144 (5′-uaCAGUAUAGAUGAUGUAcuag-3′) was synthesized by Eurofins Genomics AT GmbH (Vienna, Austria). The modifications include 3′-cholesterol, 3 × PTO (phosphorothioate) 3′-side, 2 × PTO 5′-side, and 2′-O-methyl-RNA.

### Animal experiments

Animal protocols were approved by the Institutional Animal Care and Use Committees of the Cincinnati Children’s Hospital Medical Center in accordance with the Animal Welfare Act (AWA) and PHS Policy on Humane Care and Use of Laboratory Animals. Mice were housed in a fully equipped animal facility, with free access to food and water ad libitum, and on a 12 h dark/light cycle. For miR-144 KO mouse functional characterization, both male miR-144 KO mice and WT littermates underwent echocardiography at age 8, 12, 24, and 48 weeks and their body weights were recorded before echo scans for growth comparison. MI was induced in 8–10 week old miR-144 KO mice and WT littermates (see below for details) via ligation of the left anterior descending coronary artery. Deaths were recorded and animals autopsied for occurrence of cardiac rupture. Cardiac function was evaluated via echocardiography 4 weeks post MI. The animals were then sacrificed and the hearts harvested. Each heart was washed with PBS, weighed, sliced into 5 pieces transversely, and processed for PicoSirus Red staining for infarction size and scar thickness. Hearts from another set of animals underwent the same procedure and were processed for quantification of hydroxyproline and pyridinolines. For LOX1 and zinc finger E-box binding homeobox 1 (Zeb1) analysis, mice were sacrificed 7 days after MI or sham procedure.

Tail vein injection was performed as we described previously^32^. Briefly, mice were pre-warmed in housing cage with a heat lamp for 15 min to dilate the blood vessel. Each mouse was restrained in a Tailveiner Restrainer from Braintree Scientific (Braintree, MA) and its tail was swabbed with an alcohol pad to increase the visibility of the vein. Less than 200 µl of miRNA was injected with BD 1 ml syringe 27G needle. The needle was removed from the vein and slight pressure was applied to the puncture site with a piece of gauze until the bleeding stopped.

### Myocardial infarction

Eight to ten week old mice weighing 25–30 g were anesthetized with isoflurane, intubated, and ventilated with an anesthesia machine. Each mouse was placed on its right side, a thoracotomy was performed with an incision between the fourth and fifth intercostal spaces. The lungs were retracted with a small piece of gauze, the pericardium was opened, and the proximal left anterior descending coronary artery was ligated with an 8–0 nylon suture. Lungs were inflated and the thoracotomy site was closed. To better control infarction size, this procedure was performed by the same experienced, blinded, investigator.

### Echocardiography

Anesthesia was induced with inhaled isoflurane 0.8–1.5%, delivered via drop box and nose cone. Chest hair was removed with a depilatory agent, and the mouse secured with tape to the warmed imaging platform. Scans were performed using the Vevo2100 mouse echocardiography system equipped with a 40 MHz high-frequency transducer. Mice were imaged at an anesthetic depth providing a target heart rate of 400–600 beats per minute. Measurements were made offline by investigators blinded to treatments.

### Neonatal cardiac fibroblast culture

Cardiac fibroblasts were isolated as described previously^44^. Passage 3 cells were plated onto 6-well plates, starved of serum overnight, and treated with TGFβ or doxorubicin for 24 h. The cells were harvested in lysis buffer and subjected to Western blot for Zeb1 and LOX1 analysis.

### Western blot

Protein was isolated after centrifugation of homogenized tissue in a lysis buffer containing 150 mM NaCl, 50 mM Tris·Cl (pH 7.5), 0.5% deoxycholate, 0.1% SDS, 1% nonidet P-40, PhosSTOP, and Complete Mini. The protein concentration was determined with the Coomassie protein assay kit using BSA as the standard. Aliquots of samples (50 μg protein) were subjected to SDS-PAGE and electro-transferred to a PVDF membrane at 35 V overnight at 4 °C. The membrane was incubated in 5% nonfat milk in PBS containing 0.1% Tween 20 (PBS-T) for 60 min at room temperature and then overnight at 4 °C in the same buffer containing primary antibody. The membrane was washed with PBS-T and incubated with HRP-conjugated secondary antibody at room temperature for 1.5 h. After being washed, the membrane was developed with SuperSignal West Pico Chemiluminescent reagent at room temperature. The signal was detected through an exposure to x-ray film and analyzed by scanning densitometry. The target protein was normalized to the loading control which is α-tubulin.

### Collagen content and cross-linking assays

Collagen content and cross linking were represented by hydroxyproline and pyridinolines content which was determined by assay kits and expressed as percent of WT controls. For this, left ventricular myocardium was weighed and homogenized in water. An aliquot of the homogenate was hydrolyzed with concentrated HCl at 120 °C for 3 h. The hydrolyzed sample was cleared with 0.45 µm PVDF filter unit and dried. The sample was re-suspended in water for hydroxyproline and pyridinolines assays with kits following the manufacturer’s directions.

### PicroSirius Red stain

Two slides from each of the 5 slices per mouse were de-waxed, hydrated, and stained with PicroSirius red for an hour at room temperature. The slides were washed with acidified water and dehydrated with ethanol. The slides were cleared in xylene and mounted in a resinous medium. Images were captured at different magnifications in a consistent way for all the animals.

Infarction size was measured based on the images from PicroSirius red staining by using Image J software. Infarction size is expressed as percent of left ventricle circumference from average measurements of the 5 slices.

Scar thickness was also measured based on the PicroSirius red staining images using image J software. The two smallest measurements from the 5 slices was recorded as scar thickness.

### Statistical analysis

Data are expressed as means ± SE. Differences in mean values were analyzed by a two-tailed *t*-test. P values < 0.05 were considered significant.

## Electronic supplementary material

Supplementary
