# Supplementary material for: Loss of miR-144 signaling interrupts extracellular matrix remodeling after myocardial infarction leading to worsened cardiac function
Source: Sci Rep. 2018 Nov 15;8:16886. doi: 10.1038/s41598-018-35314-6 (PMC6237773; doi:10.1038/s41598-018-35314-6)
Supplement: Supplementary file 1 — Supplementary [file 41598_2018_35314_MOESM1_ESM.docx]

**Loss of miR-144 signaling interrupts extracellular matrix remodeling after myocardial infarction leading to worsened cardiac function**

Quan He*^1^*, Fangfei Wang*^1^*, Takashi Honda*^1^*, Jeanne James*^1^*, Jing Li*^2^* and Andrew Redington*^1*^*

*^1^*The Heart Institute, Cincinnati Children’s Hospital Medical Center, Cincinnati, Ohio, USA

*^2^*Division of Cardiology, Labatt Family Heart Center, Hospital for Sick Children, Toronto, Ontario, Canada

*Address correspondence to this author at 3333 Burnet Ave., the Heart Institute, Cincinnati Children’s Hospital Medical Center, OH45229; Tel: 513-636-3049; Fax: 513-636-7117: Email: [Andrew.redington@cchmc.org](mailto:Andrew.redington@cchmc.org)

## Supplementary figure 1

Full-length blots for Fig. 4a


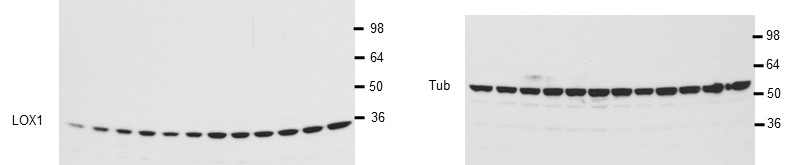


The blot was probed with LOX1, stripped and re-probed with α-tubulin.

Full-length blots for Fig. 4b


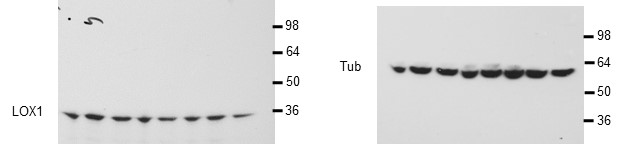


The blot was probed with LOX1, stripped and re-probed with α-tubulin.

Full-length blots for Fig. 4c


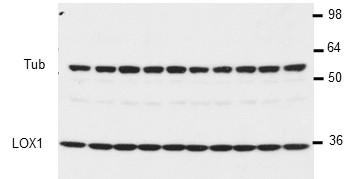


The blot was separated into two pieces between pre-stained protein marker 36 and 50 kD. The two parts were probed with LOX1 and tubulin concurrently, and exposed together.

## Supplementary figure 2

Full-length blots for Fig. 5b


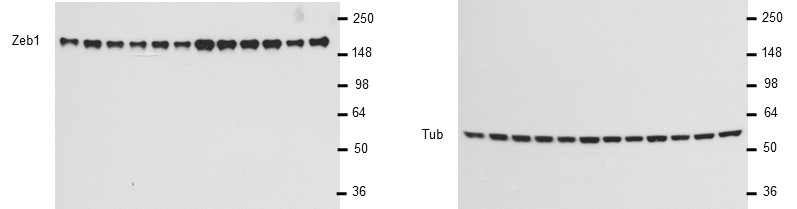


The blot was probed with Zeb1, stripped, and re-probed with tubulin.

Full-length blots for Fig. 5c


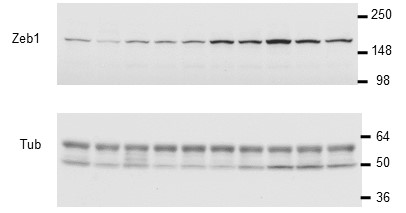


The blot was separated into two pieces between pre-stained protein marker 64 and 98 kD. The two parts were probed with Zeb1 and tubulin concurrently, and exposed separately.

Full-length blots for Fig. 5d


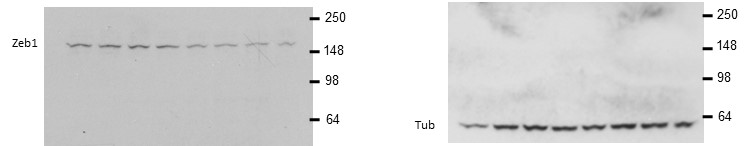


The blot was probed with Zeb1, stripped, and re-probed with tubulin.

## Supplementary figure 3

Full-length blots for Fig. 6a


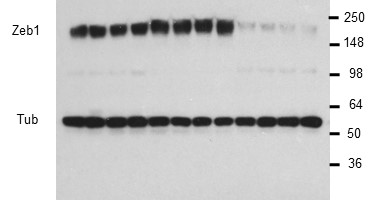


The blot was separated into two pieces around pre-stained protein marker 98 kD. The two parts were probed with Zeb1 and tubulin concurrently, and exposed together.

Full-length blots for Fig. 6b


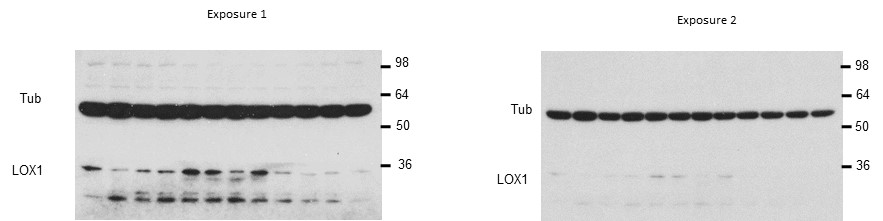


The blot was separated into two pieces between pre-stained protein marker 36 and 50 kD. The two parts were probed with LOX1 and tubulin concurrently, and exposed together. LOX1 from exposure 1 and tubulin from exposure 2 were chosen for representative picture presented in Fig. 6B.
